# To diversify or not to diversify, that is the question. Pursuing agricultural development for smallholder farmers in marginal areas of Ghana

**DOI:** 10.1016/j.worlddev.2019.104682

**Published:** 2020-01

**Authors:** Mauricio R. Bellon, Bekele Hundie Kotu, Carlo Azzarri, Francesco Caracciolo

**Affiliations:** a Comisión Nacional para el Conocimiento y Uso de la Biodiversidad (CONABIO), Liga Periférico-Insurgentes Sur No. 4903, Tlalpan, Mexico City 14010, Mexico; b International Institute of Tropical Agriculture, TL 06 Tamale, Ghana; c International Food Policy Research Institute, Washington D.C., United States; d Department of Agricultural Sciences, University of Naples Federico II, Via Universita 96, 80055 Portici (Na), Italy

**Keywords:** Crop diversity, Production diversification, Agricultural development, Ghana

## Abstract

Many smallholder farmers in developing countries grow multiple crop species on their farms, maintaining *de facto* crop diversity. Rarely do agricultural development strategies consider this crop diversity as an entry point for fostering agricultural innovation. This paper presents a case study, from an agricultural research-for-development project in northern Ghana, which examines the relationship between crop diversity and self-consumption of food crops, and cash income from crops sold by smallholder farmers in the target areas. By testing the presence and direction of these relationships, it is possible to assess whether smallholder farmers may benefit more from a diversification or a specialization agricultural development strategy for improving their livelihoods. Based on a household survey of 637 randomly selected households, we calculated crop diversity as well as its contribution to self-consumption (measured as imputed monetary value) and to cash income for each household. With these data we estimated a system of three simultaneous equations. Results show that households maintained high levels of crop diversity: up to eight crops grown, with an-average of 3.2 per household, and with less than 5% having a null or very low level of crop diversity. The value of crop species used for self-consumption was on average 55% higher than that of crop sales. Regression results show that crop diversity is positively associated with self-consumption of food crops, and cash income from crops sold. This finding suggests that increasing crop diversity opens market opportunities for households, while still contributing to self-consumption. Given these findings, crop diversification seems to be more beneficial to these farmers than specialization. For these diversified farmers, or others in similar contexts, interventions that assess and build on their *de facto* crop diversity are probably more likely to be successful.

## Highlights

• Smallholders in northern Ghana maintain high levels of *de facto* crop diversity.
• Most species are used for both self-consumption and market sales.
• The value of species used for self-consumption was on average 55% higher than that of crop sales.
• Crop diversity is positively associated with self-consumption of food crops, and cash income from crops sold.
• Crop diversification seems more beneficial to the farmers in northern Ghana than specialization.

## 1 Introduction

The conventional narrative of agricultural development, referred here as market-based agricultural specialization, foresees a pathway of increased specialization at the farm level associated with enhanced market participation (Timmer, 1997). Supporting this process has been an important objective of agricultural policies in developing countries for many decades, particularly under the Green Revolution (Evenson & Gollin, 2003). An alternative narrative, referred to here as market-based agricultural diversification, foresees a shift away from monoculture, towards a variety of crops to meet market demand at different times of the year, eventually leading to a shift of resources from one crop to a broader mix of crops and/or livestock with the aim to increase household income and profit (Asante et al., 2018, Petit and Barghouti, 1992). Both narratives are grounded on a strong participation of farmers in markets (Emran & Shilpi, 2012). It is well established however that while markets for inputs and outputs are widespread in most rural areas of the developing world, particularly in Sub-Saharan Africa, and most rural households participate in them both as consumers and producers (Barrett, 2008, Carletto et al., 2017, Chamberlin and Jayne, 2013), markets generally tend to function poorly (Barrett, 2008, de Janvry et al., 1991, Dillon and Barret, 2017, Key et al., 2000). Prices are therefore a misleading signal of value, and profitability can then be a distorted information source for understanding smallholder farmerś agricultural decisions and their resulting welfare outcomes. Therefore, in an increasingly complex farming system and incentive landscape, failure to take into account economic factors, such as self-consumption, cultural values and preferences, is inadequate. These complex incentives underpin the strategy of many smallholder farmers in developing countries who continue to grow multiple crop species in an integrated farming system, maintaining *de facto* crop diversity.

Maintaining on farm crop diversity has usually been associated with risk-coping strategies (Asfaw et al., 2019, Di Falco and Chavas, 2009, Di Falco and Perrings, 2005, Lin, 2011, McCord et al., 2015); however, there is increasing recognition of other benefits as well, particularly under poorly functioning markets. These benefits include optimizing crop production under heterogeneous agro-ecological conditions in rainfed marginal areas (Benin et al., 2004, Di Falco and Chavas, 2009, Kawa et al., 2015); producing a variety of products for different uses (Keleman et al., 2013, King, 2007); providing commercial opportunities in multiple local markets (Devaux et al., 2009, McCord et al., 2015); as well as reducing vulnerability to market and climate variability (Benin et al., 2004, Lin, 2011, McCord et al., 2015). There is increasing evidence of the positive contribution of crop diversity to household dietary diversity (Bellon et al., 2016, Dillon et al., 2015, Jones et al., 2014, Powell et al., 2015, Remans et al., 2011, Sibhatu et al., 2015). Crop diversity is also associated with self-consumption of crops with higher nutritional content, quality, and cultural significance (Frison et al., 2006, Hoffman and Mwithirwa-Gatobu, 2016), complementing foods purchased in markets (Bellon et al., 2016). It also facilitates adaptation to climate change (Douxchamps et al., 2016, Lin, 2011) and poverty reduction (Michler & Josephson, 2017).

Policy interventions associated with agricultural development have rarely taken into consideration the *de facto* crop diversity maintained on farm by smallholder farmers as an entry point for fostering agricultural innovation. This is an area that merits further attention due to the increased recognition of the multiple benefits that crop diversity can contribute to smallholder farmerś wellbeing. Successful interventions address the needs and priorities of their target beneficiaries. Therefore, if crop diversity is an important and valuable component of smallholderś farming systems and livelihoods, incorporating crop diversity in the development of interventions becomes crucial for improving their wellbeing (Bellon, Gotor, & Caracciolo, 2015).

This paper examines the relationship of crop diversity to: (i) the contribution of food crops to self-consumption; and (ii) the cash income derived from crops sold, of smallholder farmers in areas targeted for the implementation of an agricultural research-for-development project in northern Ghana. These two variables directly impact household wellbeing. The former directly affects food consumption, dietary diversity, and food security. The latter influences the ability to purchase goods and services in the market, and thus bears an indirect effect on food consumed, dietary diversity and food security, as well as in other dimensions of wellbeing such as health, housing, and education. Crop diversity, however, could relate differently in magnitude and direction to self-consumption and cash income,^1^ with different implications for agricultural development strategies. The same direction of the relationships between crop diversity and each of the variables of interest would indicate that the same strategy (diversification or specialization) is functional for both outcomes, while divergent relationships would show that these strategies are in conflict. By examining the existence and direction of these relationships, it would be possible to assess whether smallholder farmers in Northern Ghana, or in similar settings, may benefit more from interventions more focused on an agricultural diversification or a specialization strategy.

The hypothesis tested here is that for farmers under the agro-ecological and socioeconomic conditions typical of Northern Ghana and also quite common in many other regions of Africa as well as Asia, characterized by poor market development and high environmental heterogeneity in terms of soils, topography, and climate variability, an agricultural diversification rather than a specialization strategy is more beneficial. The present study is based on data collected under the Africa Research In Sustainable Intensification for the Next Generation (Africa RISING) program. This is an innovative research-for-development program that is being implemented in Ghana, Mali, Ethiopia, Tanzania, Malawi, and Zambia since 2012. As part of this initiative, a project has been carried out in the Northern, Upper West, and Upper East regions of Ghana, our areas of focus.

The remainder of the paper is organized as follows: In Section 2, the study area, data collection process and methodology employed are described. Section 3 reports the estimates of the empirical models used to assess the relationship between crop diversity and household variables of interest. Section 4 discusses the results; and finally Section 5 presents the conclusions.

## 2 Data and methods

### 2.1 Study area

**Fig. 1:**
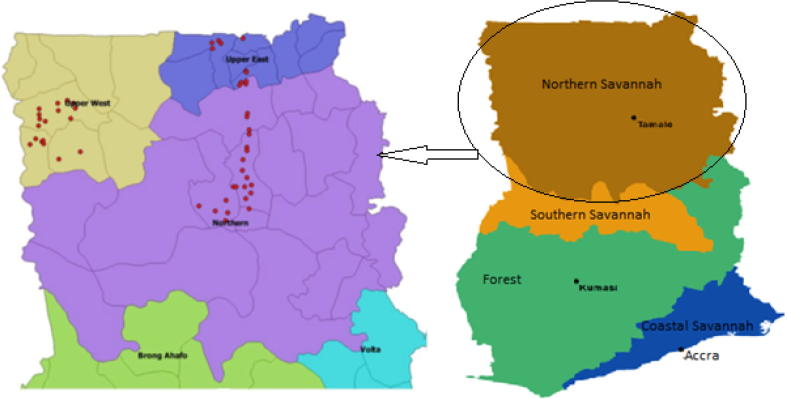
Location of the study areas in Ghana (adapted from Nin-Pratt and McBride (2014) and Signorelli et al. (2017)).

Ghana can be divided into four agro-ecological zones: the Coastal Savannah, the Forest Zone (constituting both the rain forest and the deciduous forest areas), the Southern Savannah (the transitional zone), and the Northern Savannah (Nin-Pratt & McBride, 2014) (Fig. 1). The Coastal Savannah, the Forest Zone, and Southern Savannah form the southern half of Ghana while the Northern Savannah forms the northern half of the country. Annual rainfall decreases from about 2200 mm to about 900 mm as one moves from the southwest tip to the northeast tip of the country (MoFA, 2017). The anomaly in this general agro-ecological pattern is the Coastal Savannah which receives only 800 mm of rainfall per annum. The three southern agro-ecological zones are characterized by a bimodal rainfall which allows two cropping seasons, while the Northern Savannah shows a unimodal rainfall zone. The Northern Savannah is characterized by cereal-legume based farming systems while the southern agro-ecologies are dominated by perennial food and cash crops such as plantain, cassava, cocoa, yam, and cocoyam. The rainfall distribution in the Northern Savannah is erratic causing crop failures, while being relatively stable in the southern agro-ecologies. Furthermore, rural livelihoods are more vulnerable to drought and other climatic shocks, and poverty is more pervasive in the Northern Savannah than in the southern agro-ecologies (Kotu et al., 2017, Nin-Pratt and McBride, 2014).

Our study regions are located in the Northern Savannah. While these areas show generally similar farming systems, they vary in terms of rainfall distribution, cropping patterns and socioeconomic settings. The Northern and the Upper West regions receive about 1100 mm of annual rainfall whereas the Upper East Region receives a lower amount (about 900 mm per annum) (MoFA, 2017). Maize, groundnut, rice, sorghum, millet, and cowpea are cultivated in the three regions while various livestock types such as goats, sheep, cattle, and pigs are reared (Ellis-Jones et al., 2012, Kotu et al., 2017). More drought-tolerant crops such as sorghum and millet are dominant in the Upper East Region because of its unpredictable rainfall distribution. Maize is the most dominant crop in the Northern and Upper West regions although recent trends show that its cultivation is rapidly growing in Upper East Region replacing sorghum and millet (Ellis-Jones et al., 2012). Farmers produce crops and livestock for self-consumption as well as for sale in all regions. The Upper East Region shows a high rural population density (118 persons/km^2^) relative to the Northern Region (35 persons/km2) and the Upper West Region (38 persons/km^2^) with consequent higher scarcity of farmland in the former (MoFA, 2017).

### 2.2 Data

Data were collected as part of the Ghana Africa RISING Baseline Evaluation Survey (GARBES) (Tinonin et al., 2016). The GARBES data reflect the design of the Africa RISING program implementation, based on a quasi-randomized control trial methodology. Districts in three regions were classified into six unique classes (domains) based on a combination of length of growing period and market access as proxies for agricultural potential and socio-economic integration in food value chains. A total of 50 communities were selected, with 25 interventions and 25 control communities. The sampling strategy followed a stratified two-stage random sampling approach. The first stage consisted of the purposeful selection of the intervention communities (treated) based on their biophysical characteristics and market access and then a random selection of communities (control) not exposed to the program but belonging to the similar domain as the former to obtain a suitable counterfactual. In the second stage, in control communities, 20 households per community were randomly selected; while in intervention communities, 8 randomly-selected households were included in addition to participating households who were not randomly selected. For the latter, since selection requirements are unknown, they were excluded from the sample used in this study.^2^ Our sample included 637 randomly selected households, comprising 464 from control communities and 173 from intervention communities.

The multi-topic household and community questionnaires collected information on general socio-economic characteristics, food security, poverty, nutritional status, as well as agricultural production and productivity. In particular, the survey collected information on a predetermined list of 44 specific species of crops and trees.^3^ Data are available for 23 food crops, one fruit tree (mango), two industrial crops (cotton and tobacco) as well as six general categories (without specifying the species) of “other” cereals, pulses and nuts, vegetables, roots and tubers, perennial, and other crops.^4^ The survey recorded the quantity harvested for each crop in the farm and the quantities allocated to seed, self-consumption, sale, exchange and gifts, as well as animal feed and fodder.

For the quantities sold, the price received per kg of produce was recorded, as well as an estimated total value for gifts, exchange, saved for seed, and animal feed. Our analysis focused on the value of the quantities used for self-consumption and for direct sales. No shadow prices were collected for self-consumption; hence the median of the purchased price/kg for a crop in each of the 50 communities was multiplied by the quantity kept for self-consumption by each household to estimate the imputed monetary value of a crop kept for self-consumption, as proposed by Deaton and Zaidi (2002). If no specific price was available for a particular crop in a village, the median price at a progressively higher geographical level was employed. In the case of gross income derived from crop sales, the median price/kg per crop in a community was used, since some variation in the collected prices occurred at the household level.

For the portfolio of species grown by a household, area planted with each species was collected for the last completed season. A Simpson diversity index (SDI) of the crops grown was calculated for each household based on the share of area planted with each species^5^ (Smale, 2006):

(1) $$\mathrm{SD}I_{i}=1-\sum \alpha_{\mathrm{ij}}^{2}$$

where *α_ij_* is the area share occupied by the *j*-species grown by household *i.*

The SDI combines indicators of crop richness and abundance. A high score will indicate not only that there are many species in the farm, but also that they are distributed evenly across the farmed area. A zero score will indicate that one species occupies the whole farmed area, i.e. complete specialization. Furthermore, this index is equal to one minus the Herfindahl index—an index used in the economic literature to measure concentration in any specific industry (Meng, Smale, Bellon, & Grimanelli, 1998). Hence, the SDI is well suited to analyze the diversification-specialization nexus at the crop species level in a farming system.

The survey provided detailed information on: (1) area planted and total labor inputs for each crop grown in the last completed cropping season; (2) quantity and value of purchased inputs; (3) housing conditions, number of durable agricultural and non-agricultural assets, livestock, and land of households, which were used to derive a wealth index through factor analysis (Filmer and Pritchett, 2001, Hong et al., 2006). Based on the location of each community, data on climatic variability (interpolation of observed data over the period 1950–2000) were extracted from WorldClim (http://worldclim.org/). Rural population density (people/km^2^) and travel time (minutes) to nearest town over 50,000 people were sourced from IFPRI-HarvestChoice (http://harvestchoice.org). Variables on climatic variability included: (a) precipitation seasonality, which is a measure of the variation in monthly precipitation totals over the course of the year, and (b) isothermality, that quantifies how large the day-to-night temperatures oscillate relative to the summer-to-winter (annual) oscillations.

### 2.3 Modeling approach

This paper aims to investigate if crop diversification strategy employed by smallholder farmers is associated with household food self-consumption—measured as the imputed value derived from the purchase price times the quantity of crops allocated to self-consumption—and cash income based on market sales. This research question can be methodologically answered through a system of three simultaneous equations—one for each of the three domains of interest, namely crop diversity, household food self-consumption and cash income, in order to identify their determinants and interrelations.

The stochastic version of the empirical model is formulated for the *i*-th household as:

(2) $$\mathrm{Crop}\;Diversity_{i}=x\prime_{\text{i}}\beta +z\prime_{\text{i}}\gamma +{\text{e}}_{i}$$

(3) $$\mathrm{Log}\;Agr.\;Cash\;Income_{i}=x\prime_{\text{i}}\lambda +\delta \;Crop\;Diversity_{\text{i}}+u_{i}$$

(4) $$\mathrm{Log}\;Agr.\;Self\;\text{-}\;Consumption_{i}=x\prime_{\text{i}}\theta +\xi \;Crop\;Diversity_{\text{i}}+\varepsilon_{i}$$

Eq. (2) models crop diversity (in terms of SDI of the crops grown) by each household based on the share of area planted to each species as a linear function of explanatory factors **x** related to household characteristics,^6^ labor, other inputs, access to markets, climate and location. These variables control for regional, household and land heterogeneity that potentially could confound the effect of crop diversity on agricultural cash income and self-consumption, i.e. if omitted they could bias the estimates of the relationships of interest; the variables included have been commonly used in other studies on crop diversification (e.g., Asfaw et al., 2019, Bellon et al., 2015, Bellon et al., 2016, Benin et al., 2004, Michler and Josephson, 2017, Van Dusen and Taylor, 2005). Eqs. (3) and (4) model, respectively, the natural logarithm of household cash income derived from crop sales and the natural logarithm of the imputed value from food crops destined for direct household consumption, as a function of crop diversity and the same explanatory factors as in Eq. (2).

Instrumental variables were used since endogeneity between crop diversity and the two variables of interest might be affecting the parameter estimates. Distance to the city proxied by travel time to city with at least 50,000 inhabitants and the use of extension services were used as excluded instruments (Eq. (2), vector **z***_i_*). Similar instruments were already proposed in other recent studies of household adaptation strategies (such as crop diversification) and livelihood outcomes.^7^ The instruments were assumed to influence crop diversity, without exerting any “direct” effect on the other two endogenous variables. The specification passed both the Hansen-Sargan test of over-identifying restrictions and the test for weak instruments carried out to test for the validity and relevance of the instruments. The parameters of the instruments (*δ* and *ξ*) and the other parameters of the system of equations (**β**, **γ**, **λ** and **θ**) were jointly estimated using the Three Stage Least Squares (3SLS) procedure. All statistical analyses were performed using STATA software (Version 15.1, http://www.stata.com).

## 3 Results

### 3.1 Crop diversity, self-consumption and income among smallholder farmers

**Fig. 2:**
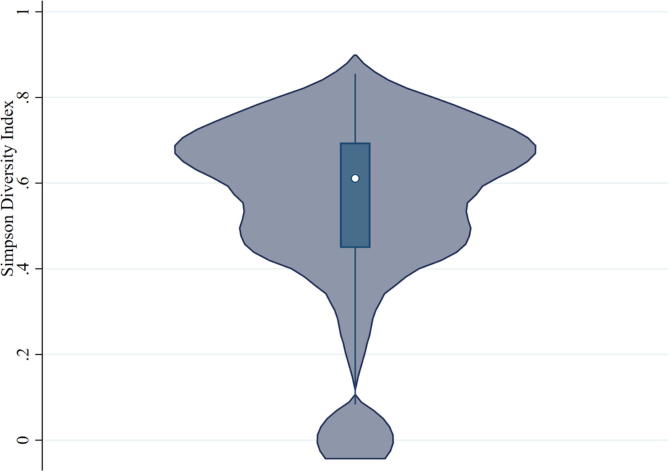
Violin plot of Simpson diversity index.

Smallholder farmers in our sample reported on average 3.2 (±1.4) crops per farm and an SDI of 0.54 (±0.2) (Table 1). The violin plot of the SDI shows median, quantiles and a skewed distribution toward high levels of crop diversity (Fig. 2), indicating that on average these farmers tend to grow a relatively high number of crops, with less than 5% of them characterized by a null or very low level of crop diversity (SDI < 0.2). The average imputed value from the quantities used for self-consumption was GH₵ 857.79 (±755.41) and the average gross income from crop sales was GH₵ 554.02 (±988.42), which taken together sum up to the overall income derived from agricultural production per household, with a mean of GH₵ 1411.82 (±1395.43).^8^ Table 2 shows the crops grown in the study area, the number of households who use each of them for self-consumption and for sale, their aggregate and average value (imputed for self-consumption). Most species are used for both self-consumption and sale, while four of them are dominant in both uses: maize, rice, groundnut and yams. Even for these species, the imputed value of self-consumption is much larger than the income from their sale, particularly for maize and rice. Although maize is clearly dominant, the fact that the other main crops are used by less than a third of households combined with the skewed distribution of the SDI indicates a notable diversity at the farm level. Furthermore, the imputed value of self-consumption is on average higher by 55% than that of crop sales.

**Table 1:** Indicators of crop diversity and income.

Variable	Mean	St. Dev.	Min	Max
Number of crops	3.2	1.4	1	8
Simpson diversity index	0.54	0.2	0	0.86
Imputed value from self-consumed crops (GH₵)	857.79	755.41	0	7735.12
Gross income from crops sold (GH₵)	554.02	988.42	0	11537.50
Total value of agricultural production (GH₵)	1411.82	1395.43	8.55	12751.11

*Source:* Data from the Ghana Africa RISING Baseline Evaluation Survey – 2014 (Tinonin et al., 2016)

**Table 2:** Plant species grown by farm households in North Ghana and their associated value of (imputed) self-consumption and sales (GH₵).

Crop	Self-consumption				Sale			
	No. hh	%	Sum	Average/hh	No. hh	%	Sum	Average/hh
*Cereals*								
Maize	554	81.2	199522.33	360.15	197	28.9	86109.94	437.11
Pearl millet	151	22.1	23229.24	153.84	48	7.0	10698.53	222.89
Sorghum	70	10.3	9472.88	135.33	21	3.1	6806.23	324.11
Finger millet	52	7.6	7812.41	150.24	11	1.6	2560.75	232.80
Rice	222	32.6	88975.53	400.79	144	21.1	58849.86	408.68
Other cereals	1	0.1	46.30	46.30	0			

*Pulses & nuts*								
Bean	164	24.0	31149.4	45.7	50	7.3	10514.90	210.30
Soybean	82	12.0	19278.1	28.3	84	12.3	37648.19	448.19
Pigeon pea	5	0.7	1592.6	2.3	4	0.6	391.00	97.75
Chickpea	8	1.2	1942.0	2.8	6	0.9	1429.50	238.25
Cowpea	27	4.0	5911.4	8.7	17	2.5	20934.25	1231.43
Groundnut	206	30.2	99725.9	146.2	180	26.4	66039.84	366.89
Bambara nut	94	13.8	11613.5	17.0	24	3.5	4842.58	201.77

*Vegetables*								
Okra	3	0.4	459.38	153.13	2	0.3	2232.00	1116.00

*Roots & tubers*								
Onion	1	0.1	200.00	200.00	1	0.1	4900.00	4900.00
Potato	1	0.1	41.67	41.67	1	0.1	400.00	400.00
Sweet potato	3	0.4	150.00	50.00	2	0.3	950.00	475.00
Cassava	17	2.5	5348.39	314.61	9	1.3	2726.25	302.92
Yam	145	21.3	69756.27	481.08	67	9.8	34882.29	520.63
Total	633	92.8	546454.10	863.28	444	65.1	352916.11	794.86

*Source:* Data from the Ghana Africa RISING Baseline Evaluation Survey – 2014 (Tinonin et al., 2016).

### 3.2 Econometric results

**Fig. 3:**
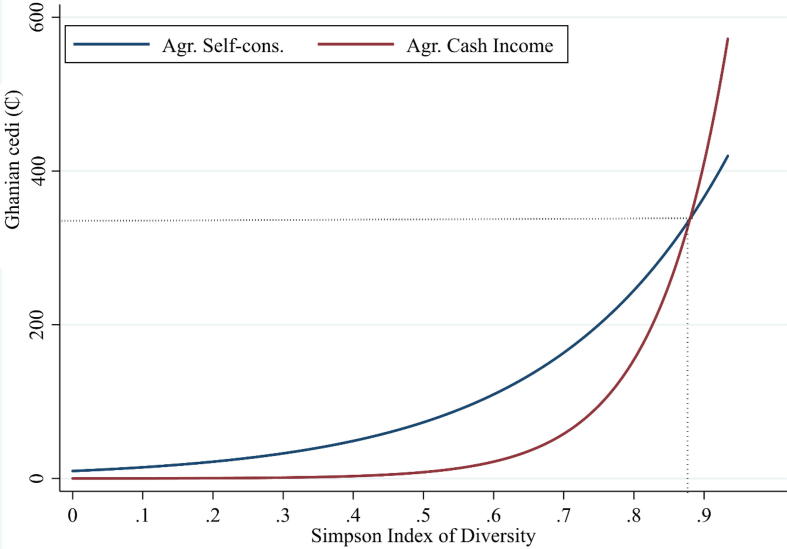
Predicted relationship between crop diversity and the value of agricultural sales as well as self-consumption.

Table 3 shows a description and descriptive statistics of the explanatory factors included in the system of three simultaneous equations, while Table 4 shows the empirical econometric results. The latter show that (1) a more diverse crop portfolio is significantly and positively associated to both cash income (*δ* = 0.425; p = 0.027) and imputed value from self-consumption (*ξ =* 0.175; p = 0.010). Thus, crop diversification seems to be more beneficial to these famers than specialization, for both our variables of interest. However, the size and significance of the coefficients show that crop diversity is better correlated with cash income from sales than with the amount of food crops destined for self-consumption. Fig. 3 conveys a similar finding showing that for low levels of crop diversity the value of self-consumption dominates over the value of agricultural sales, but for high levels of crop diversity, the opposite happens. Therefore, increasing crop diversity seems to open market opportunities for farmers, while still providing a substantial contribution to self-consumption.

**Table 3:** Definition and descriptive statistics of variables used in the regression model.

Variable name	Description	Mean	Std.dev.	Min	Max
Sex of head of household	Dummy variable: 1 = male, 0 = female	0.91		0	1
Age of head of household	Years of age	46.97	15.31	18	91
Education head of household	Completed years of formal education by household head	2.30	4.36	0	16
Family size	Number of household members	8.27	5.45	1	40
Dependency ratio	Ratio of the number of household members age ≤14 and > 64 to those age 15–64 years old	1.12	0.77	0	5
Receiving advice/information from extension	Dummy variable: 1 = household received advice/information from agricultural development/extension agent in the last 12 months; 0 = otherwise	0.49		0	1
Area planted	Number of hectares planted by a household (ha)	3.01	2.13	0.2	14.2
Number of parcels	Number of different parcels farmed by a household	2.35	1.16	1	9
Wealth Index	Asset index constructed using factor analysis (principal-component factor method) based on the predicted value of the first factor in the principal component based on housing/dwelling conditions, number of asset durables (agricultural and non-agricultural), livestock, as well as land ownership^1^	0.01	1.16	−1.50	9.25
Wealth Index squared	Squared of the wealth index	1.34	5.91	4.5e-06	85.57
Total labor	Number of persons-day invested in agricultural production in previous 12 month period	244.58	169.51	7	1427.5
Treatment community	Dummy variable: 1 = household selected from a treatment community; 0 = household selected from a control community	0.27		0	1
Travel time to city with 50,000 inhabitants	Hours of travel to the nearest city with 50,000 inhabitants	1.92	0.76	0.56	5.40
Rural population density	Number of persons living in rural areas/km2	56.87	40.19	22.2	248.6
Isothermality	Quantifies how large the day-to-night temperatures oscillate relative to the summer-to-winter (annual) oscillations (ratio)	59.82	0.26	59.00	60.19
Precipitation seasonality	Measure of the variation in monthly precipitation totals over the course of the year. It is the ratio of the standard deviation of the monthly total precipitation to the mean monthly total precipitation (also known as the coefficient of variation, expressed as a percentage).	101.69	6.31	90.89	111.68
Northern Region	Dummy variable: 1 = household located in the Northern Region; 0 = otherwise	0.52		0	1
Upper East Region	Dummy variable: 1 = household located in the Upper East Region; 0 = otherwise	0.12		0	1
Upper West Region	Dummy variable: 1 = household located in the Upper West Region; 0 = otherwise	0.36		0	1

1 Weights assigned to each element in the index were obtained according to the methodology proposed by Filmer and Pritchett (2001).

**Table 4:** System of simultaneous equations modeling crop diversity, agricultural income from sales, and imputed self-consumption.

	Crop diversity^1^			log (total ag. sales)			log (total ag. Self cons)		
	Coeff	std.err	*p*.value	Coeff	std.err	*p*.value	Coeff	std.err	*p*.value
Crop diversity^1^				**0.425**	**0.192**	**0.027**	**0.175**	**0.068**	**0.010**
Sex of head of household (Dummy variable 1 = male)	0.338	0.256	0.186	**−0.336**	**0.188**	**0.073**	0.082	0.066	0.216
Age of head of household	−0.007	0.005	0.169	**−0.010**	**0.004**	**0.006**	0.001	0.001	0.422
Education head of household	0.001	0.017	0.939	**−0.023**	**0.012**	**0.058**	**−0.008**	**0.004**	**0.070**
Family size	**−0.061**	**0.019**	**0.001**	−0.014	0.018	0.431	**0.011**	**0.006**	**0.083**
Dependency ratio	**0.274**	**0.094**	**0.003**	−0.136	0.082	0.100	−0.011	0.029	0.708
Area planted	**0.184**	**0.048**	**0.000**	**0.132**	**0.049**	**0.007**	0.028	0.017	0.105
Number of parcels the household farms	**0.254**	**0.069**	**0.000**	**−0.132**	**0.072**	**0.064**	−0.035	0.025	0.162
Wealth index	**−0.211**	**0.120**	**0.078**	**0.252**	**0.086**	**0.003**	**0.124**	**0.030**	**0.000**
Wealth index squared	0.008	0.019	0.671	**−0.023**	**0.013**	**0.078**	**−0.018**	**0.005**	**0.000**
Total labor invested man-days	**0.005**	**0.001**	**0.000**	−0.001	0.001	0.158	0.000	0.000	0.335
Treatment community	**0.349**	**0.176**	**0.048**	−0.152	0.145	0.294	0.020	0.051	0.691
Rural population density	−0.001	0.002	0.707	0.002	0.002	0.168	0.000	0.001	0.808
Dummy variable, 1 = Northern region	**−1.051**	**0.223**	**0.000**	**0.423**	**0.255**	**0.097**	0.095	0.090	0.292
Dummy variable, 1 = Upper East Region	0.200	0.387	0.606	**−0.729**	**0.275**	**0.008**	−0.150	0.097	0.121
Precipitation seasonality (coefficient of variation)	**−0.036**	**0.018**	**0.039**	−0.018	0.014	0.197	0.005	0.005	0.350
Isothermality	**0.811**	**0.357**	**0.023**	0.055	0.299	0.855	**−0.263**	**0.105**	**0.012**
Dummy variable, 1 = household received advice/information from extension	**0.462**	**0.142**	**0.001**						
Travel time to city with 50,000 inhabitants	**0.224**	**0.091**	**0.013**						
Cons	**−41.664**	**20.889**	**0.046**	−0.788	16.884	0.963	**16.981**	**5.947**	**0.004**

No. Obs = 637.

*Source:* Data from the Ghana Africa RISING Baseline Evaluation Survey – 2014 (Tinonin et al., 2016).

Significance of bold values <0.1.

1 Simpson Diversity Index based on the area planted with different crops × 10.

Estimates of crop diversity equation (Eq. (2)) provide additional insights on the factors correlated with farmer’s choice of production diversification. For instance, households with more members and/or those more wealth-endowed^9^ seem to adopt a more specialized strategy with lower crop diversity. On the contrary, dependency ratio and labor invested are positively associated with crop diversity. In addition, farmers exposed to extension services are more likely to diversify than those who did not receive agricultural advice. Increased travel time to the city is also positively related to crop diversity, possibly suggesting crop diversification as a subsistence strategy for remote households probably due to high transaction costs in accessing the market. Climatic factors such as variation in precipitation and diurnal temperature are associated with crop diversity in a seemingly complex way. Indeed, while precipitation variability is estimated to reduce crop diversity, the opposite occurs for isothermally. Finally, specific characteristics of household head (age, gender and education) do not seem to be correlated with farm diversification choice. Factors most associated to the two variables of interest include: education of the household head, wealth, area planted, and isothermality. The validity of the overall estimates is corroborated by the Hansen-Sargan econometric test of over-identifying restrictions showing that the null hypothesis cannot be rejected, and the instruments are indeed valid. Results from the test for weak instruments show that the selected instruments, i.e., use of extension services and distance from the city, were relevant (Appendix Table A1).

## 4 Discussion

The evidence presented here shows that smallholder farmers in North Ghana maintain substantial crop diversity. Crop diversity seems to be positively associated with gross income and with the imputed value of self-consumption. Thus, crop diversity is generating important direct benefits for these farming households. In fact, our results may be conservative since there is evidence that smallholder farmers in the study area maintain more crop diversity than was documented in the GARBES survey (Bellon, Atieno, & Kotu, 2018). Furthermore, our results are in line with the empirical literature on the linkage between agricultural diversification and income among smallholder farmers (Asfaw et al., 2019, Bigsten and Tengstam, 2011, Kasem and Thapa, 2011, Michler and Josephson, 2017, Nguyen, 2017), particularly those related to economies of scale/scope (Nguyen, 2017, Paul and Nehring, 2005, Rahman, 2009). Several studies also show that agricultural diversification would enhance household food/nutrition security (Bellon et al., 2016, Ecker, 2018, Hirvonen and Hoddinott, 2014, Jones et al., 2014, Sibhatu et al., 2015), something our findings also support. This is due to the fact that smallholder farmers produce crops not only for sale but, often more importantly, also for home consumption, and thus a more diversified production implies better access to higher food diversity (Ickowitz, Powell, Roland, Jones, & Sutherland, 2019). Surprisingly, precipitation variability is associated with a reduction in crop diversity, while one would expect the opposite (Di Falco and Chavas, 2009, Di Falco and Perrings, 2005). However, most of the evidence of this positive relationship refers to crop infra-specific diversity, i.e. planting multiple varieties of the same crop (e.g. Di Falco and Perrings, 2005, Di Falco and Chavas, 2009) while here it refers to planting different crops (inter-specific diversity). A possible explanation is that under increased rainfall variation, particularly under the risk of drought, farmers are unlikely to plant crops sensitive to drought, while more likely to plant those that are less sensitive, hence diversifying the varieties within the latter crops to minimize the chances of crop failure. The relationships among rainfall variability and infra-specific versus inter-specific diversity deserve further research.

Adopting a crop diversification strategy is a rational behavior for the farmers operating in the contexts of Northern Ghana. They need to adapt to relatively high environmental heterogeneity in terms of soils and topography that might create multiple production niches. For example, being highly dependent on rainfall, they cope with production risks in the face of rainfall variability. Due to poor infrastructure, they face high transaction costs to participate in input and output markets as producers, and as consumers they have high incentives for self-provision of crops and their derived products (Bezabih and Sarr, 2012, Di Falco and Chavas, 2009, Dillon et al., 2015, Kasem and Thapa, 2011, Nguyen, 2017, Rahman, 2009). Furthermore, their average farm size is too small to reap the benefits of economies of scale necessary to benefit from specialization (Jayne, Mather, & Mghenyi, 2010).

Although our results refer to a case study of the target area of an agricultural research-for-development project, and cannot necessarily be generalized to broader settings, they bear implications for the suitability of market-based agricultural specialization versus diversification strategies. A specialization pathway could be very sensitive to: (i) adverse environmental fluctuations that may reduce yields of key species produced; (ii) low diversity of foods available in the market; (iii) high market transaction costs; and (iv) substantial price fluctuations of the key species produced relative to those of the diverse foods and products they need to buy. The pathway above does not seem suitable for these farmers, particularly since it will entail the high opportunity costs of forgoing the benefits of crop diversity. Empirical studies also support diversification over specialization among smallholder farmers in Africa and elsewhere (Asfaw et al., 2019, Bezabih and Sarr, 2012, Di Falco and Chavas, 2009, Kasem and Thapa, 2011, Lin, 2011). For instance, Kasem and Thapa (2011) found that farmers who practice diversification could have continuous flow of income from crop sales since several types of crops could be harvested at different times of the year. The continuous cropping buffers price-related income shocks arising from dependence on having a few crops with similar cropping cycles. Stability in farm household income may also arise from the ability of diversified systems to suppress biotic and abiotic stresses as different crops can cope with different tolerance levels (Lin, 2011). Furthermore, the increased amount of monetary income derived from specialization may not be enough to purchase all the foods households obtain from production for self-consumption that they would forego with specialization. For example, based on the average imputed and gross monetary incomes observed among these farmers, monetary income would have to increase at least by 155% to purchase the foods produced for self-consumption.

To the contrary, a diversification pathway seems more appropriate for farmers in these settings. Given their crop diversity, this pathway may entail shifting the composition of crops grown to benefit from changing market opportunities. A slightly different approach could be to take advantage of the crop diversity already present in the farmers’ portfolio and recognize the importance of production for self-consumption. Therefore, such a pathway cannot be built on a purely market-based diversification strategy. This alternative approach should aim at strengthening the economies of scope of crop diversity that provide multiple benefits such as food for self-consumption or crop sold at the market for income generation (Ickowitz et al., 2019, Nguyen, 2017, Paul and Nehring, 2005). It should also try to lessen the negative impacts of transaction costs and adverse relative prices, while providing market opportunities due to seasonal variation and market niches (Kasem & Thapa, 2011). This approach also makes households less vulnerable to a low diversity of foods available in the market by strengthening self-consumption, which also may lessen the negative effect of food market price fluctuations (Ickowitz et al., 2019).

This paper has presented an empirical framework to test in the field the existence and direction of the relationship between *de facto* crop diversity and key variables related to farmers’ wellbeing in order to identify, without any preconceived opinion or ideological prejudice, what type of development intervention could work better. Since successful agricultural development interventions address the needs and priorities of their target beneficiaries, if crop diversity is an important component farmerś strategy to manage their agricultural systems and livelihoods then incorporating diversification into the analysis, design, and implementation of agricultural interventions becomes crucial to positively impact their well-being. The idea is to recognize the farmers’ normal behavior and the incentives they have in the specific contexts in which they operate, i.e. their natural behavior “without intervention,” in order to enhance the effectiveness of development programs. The effectiveness of diversification strategies can be influenced however, by several factors operating at the household level and beyond (Asfaw et al., 2019). Our results show that diversification is affected by demographic characteristics of farm households (family size and dependency ratio); resource endowments (land, labor and wealth); access to market and information (distance to nearest city, access to extension advice); and finally climatic factors (precipitation, temperature). Some of these factors have also been examined in other studies where most of the findings are in line with ours (Asfaw et al., 2019, Kasem and Thapa, 2011, McCord et al., 2015, Dillon et al., 2015). For instance, Kasem and Thapa (2011) show that farm households are more likely to adopt diversification strategies if they have high resource endowments (land and labor), soil suitable for different crops, good access to information through government extension services, and close interaction with farmers groups. Similarly, McCord et al. (2015) report that climatic factors, farm size, and information matter for farm households to diversify their production. These findings imply that designing a successful rural development program aiming at increasing agricultural diversification entails a careful analysis of a wide range of factors that could vary in time and space. It should be pointed out however, that a limitation of this study is that our empirical approach is based on observational cross-sectional data, with an overall relatively lower suitability to identify causal relationships, as compared to experimental data. Notwithstanding its limitation, our findings contribute to the discussion of the importance of analyses derived from associations and correlations resulting from observational data.

## 5 Conclusions

Benefits derived from market specialization have long been highlighted in the development literature, while agricultural diversification has been essentially considered a necessary cost for minimizing the risks of excessive specialization in the face of unpredictable events. Nevertheless, more recently, research and development programs have begun to focus on the advantages of crop diversity, as well as its sustainable use, as a key driver for improving livelihoods in rural communities. It is difficult to generalize however, the effectiveness of interventions based on diversification, rather than specialization, as they would largely depend on specific contexts and household characteristics. This paper proposed an empirical approach for analyzing whether farmers in specific contexts may benefit more from a diversification or a specialization agricultural strategy for managing their agricultural systems effectively and, consequently, improving their livelihoods. The approach goes beyond just the production side, incorporating consumption preferences, households, and site-specific characteristics. Results show that crop diversity at the farm level is positively associated with both self-consumption of food crops and cash income from crops sold, providing empirical evidence of the relative dominance of a diversification over a specialization strategy in this specific setting. Since successful policy needs to address the priorities of their target beneficiaries, the proposed empirical approach can be used to assess the type of interventions more likely to positively affect beneficiarieś wellbeing in specific contexts.

## Declaration of Competing Interest

The authors declare that they have no known competing financial interests or personal relationships that could have appeared to influence the work reported in this paper.

## Figures and Tables

**Table A1:** Instrumental variables: Test for instrument validity and relevance.

Hansen-Sargan overidentification statistic^1^:	0.029, *p* = 0.864
Test for weak instruments^2^:	F(2, 618) = 6.45; *p* = 0.002
Durbin–Wu–Hausman test for endogeneity^3^:	
log Cash Income	3.96 (*p* = 0.047);
log ag. Self-consumption.	2.90 (*p* = 0.089)

*Source:* Data from the Ghana Africa RISING Baseline Evaluation Survey – 2014 (Tinonin et al., 2016).

1 Small p-values indicate instrument inconsistency, H0: E(z|u) = 0.

2 Small *p*-values indicate instruments relevance, H0: E(z|x) = 0.

3 Small *p*-values indicate inconsistency of OLS, H0: E(u|x) = 0.

## Appendix A

See Table A1.

## Footnotes

1 Here we do not address the relationship between crop diversity and dietary diversity, examined under the same agriculture research project by Signorelli, Haile, and Kotu (2017).

2 The GARBES included 1284 households, of which 600 were actual or potential participants in project activities (since the survey was carried out after the project had already started). In most cases, these farmers self-selected themselves to be part of the program, given their own willingness and capacity to participate. In addition, some (randomly selected) control households reported incomplete data and therefore were excluded from the analysis.

3 The list included six species of cereal crops, eight of pulses and nuts, eleven of vegetables, seven of roots and tubers, eight of perennial crops, and four other crops. In addition for each crop category there was a category “other,” such as other cereals, other pulses and nuts, etc.

4 It should be pointed out however, that there is evidence that households in these areas maintain many more crop species than those recorded in the GARBES survey (Bellon et al., 2018).

5 The “other” categories mentioned were counted just as just one species within each category.

6 Because households selected in control and intervention communities show statistically different averages across several socioeconomic and environmental characteristics, and in order to take this source of heterogeneity into account, we included a dummy variable indicating the type of community from which the household was selected in our regression model (see below).

7 For instance, Di Falco, Veronesi, and Yesuf (2011) and Di Falco and Veronesi (2013) used extension services, while Michler and Josephson (2017) used proximity to a cooperative.

8 Since during the survey period the exchange rate averaged 2.7GH₵ per US dollar, the average annual total agricultural income corresponded to US $522.90.

9 It could be argued that crop diversity could lead farmers to enhanced wealth by increasing income and food security, while here we assume the opposite pathway. While it may be true that in the long-term crop diversity may enhance wealth (supporting diversification), in the time horizon of our study wealth is the result of past income, savings, and investments, thus being exogenous to current production and consumption decisions as well as weakly correlated to our dependent variables.
